# TIR signal promotes interactions between lipase-like proteins and ADR1-L1 receptor and ADR1-L1 oligomerization

**DOI:** 10.1093/plphys/kiab305

**Published:** 2021-07-02

**Authors:** Zhongshou Wu, Lei Tian, Xueru Liu, Yuelin Zhang, Xin Li

**Affiliations:** 1 Michael Smith Laboratories, Department of Botany, University of British Columbia, Vancouver, BC, Canada V6T 1Z4; 2 Department of Botany, University of British Columbia, Vancouver, BC, Canada V6T 1Z4

## Abstract

TIR signaling promotes the interactions between lipase-like proteins EDS1/PAD4 and ADR1-L1 immune receptor, and oligomerization of ADR1-L1.

Dear Editor,

Both plants and animals use nucleotide-binding leucine-rich repeat (NLR) immune receptors to perceive pathogens and trigger immunity. Toll/interleukin-1 receptor (TIR)-type plant NLRs (TNLs) require the lipase-like protein family members Enhanced Disease Susceptibility 1 (EDS1)/Phytoalexin Deficient 4 (PAD4)/Senescence-Associated Gene 101 (SAG101) and helper NLRs (hNLRs) for downstream signaling, the biochemical mechanisms of which remain unclear. Here, we report that TIR signaling promotes the association of EDS1 and PAD4 with hNLR Activated Disease Resistance 1-Like 1 (ADR1-L1), and the oligomerization of ADR1-L1s for downstream immune activation and cell death. 

Typical plant NLRs contain three functional domains. The C-terminal leucine-rich repeats (LRRs) are involved in effector recognition, self-repression, and protein-protein interactions. The central nucleotide-binding (NB) region serves as an oligomerization platform and ATP/ADP-binding molecular switch (Jones et al., 2016). Based on the different N-termini, plant NLRs are grouped into three main subclasses: TNLs, coiled-coil (CC)-type NLRs (CNLs), and Resistance to Powdery mildew 8 (RPW8)-like CC-type NLRs (RNLs; Jones et al., 2016). The TIR domains in TNLs function as nicotinamide adenine dinucleotide (NAD^+^) hydrolase (NADases; Horsefield et al., 2019; Wan et al., 2019). In Arabidopsis (*Arabidopsis thaliana*), there are two RNL sub-families, ADR1 and N Requirement Gene 1 (NRG1). The ADR1 family contains ADR1, ADR1-Like 1 (ADR1-L1), and ADR1-Like 2 (ADR1-L2), with redundant functions. Similarly, the NRG1 family consists of the full-length NRG1A and NRG1B protein and an N-terminally truncated NRG1C. The full-length ADR1s and NRG1s function in parallel downstream of TNLs and in basal immunity with unequal redundancy (Dong et al., 2016; Castel et al., 2019; Wu et al., 2019). Additionally, three lipase-like proteins are also required for TNL-mediated immunity, including EDS1, PAD4, and SAG101. EDS1 interacts with PAD4 or SAG101 to form distinctive EDS1-PAD4 or EDS1-SAG101 heterodimers (Wagner et al., 2013), working together with ADR1s or NRG1s genetically, forming the EDS1-PAD4-ADR1s and EDS1-SAG101-NRG1s signaling modules downstream of TNLs (Lapin et al., 2019; Wu et al., 2019). Besides TIR signaling, these two modules also contribute to plasma membrane localized receptor-mediated immunity and basal defense (Pruitt et al., 2020; Tian et al., 2020). Recently, Sun et al. (2021) reported that NRG1A/1B interact with EDS1-SAG101 dimers in an effector-dependent manner to transduce TIR signals. However, how TIR signals are transduced by EDS1/PAD4/ADR1s was unclear.

During our analysis of Arabidopsis hNLRs, we used *snc1* (*suppressor of npr1-1, constitutive 1*) and *chs3-2D* (*chilling sensitive 3, 2D*), two autoimmune TNL mutants, to establish the parallel relationships between the downstream EDS1-SAG101-NRG1 and EDS1-PAD4-ADR1 modules (Wu et al., 2019, 2021). To examine such relationship in the absence of autoimmunity, we generated higher order mutants with CRISPR/Cas9, combining loss-of-function mutations from either different or the same modules in wild-type (WT) Col-0 background (Supplemental Methods). All the newly generated mutants are indistinguishable from WT in morphology. We then challenged these plants with the avirulent bacterial pathogen *Pseudomonas syringae* pv. *tomato* (*P.s.t.*) DC3000 expressing either HopA1 or AvrRps4 effectors, which are recognized by TNL RPS6 (resistant to *P. syringae* 6; Kim et al., 2009) or RPS4 (Gassmann et al., 1999), and the virulent *P.* *syringae* pv. *maculicola* (*P.s.m.*) ES4326. Mutants from the EDS1-PAD4-ADR1 modules, including *pad4-1* and *adr1 triple*, were similarly more susceptible compared with Col-0, and combining *adr1 triple* and *pad4-c1* mutations together failed to enhance their susceptibility further (Supplemental Figure S1, A–C), supporting ADR1s and PAD4 functioning in the same genetic module. However, mutants combining mutations in components from the two different modules, including *pad4-1 sag101-1*, *pad4-1 nrg1 triple*, *sag101-c1 adr1 triple*, and *adr1 nrg1 sextuplet*, are all more susceptible, with similar pathogen growth as *eds1-2* (Supplemental Figure S1, A–C). These results corroborate that EDS1-SAG101-NRG1 and EDS1-PAD4-ADR1 function as two distinct modules downstream of TNLs.

Genetically, ADR1s function together with EDS1/PAD4 in a signaling module. However, we were not able to reproducibly detect interactions between ADR1s and EDS1/PAD4 in co-immunoprecipitation (IP) experiments. Thus, we tested whether ADR1s associate with the EDS1-PAD4 heterodimer with a split luciferase complementation (SLC) assay (Supplemental Methods). Among the three members of the ADR1 family, only ADR1-L1 expressed well and did not trigger cell death in *Nicotiana benthamiana* (Supplemental Figure S2). We therefore used ADR1-L1 in the follow-up experiments. ADR1-L1 interacted with EDS1 (Figure 1, A) and PAD4 (Figure 1, B) by SLC (Supplemental Figure S3). To confirm these interactions, we adopted the recently developed TurboID-based proximity labeling method, allowing TurboID-fused protein to biotinylate proximal and interacting proteins in the presence of biotin (Zhang et al., 2019), enabling detection of weak and transient protein-protein associations (Wu et al., 2021; Supplemental Methods). Co-expression of EDS1-ZZ-TEV-FLAG and PAD4-ZZ-TEV-FLAG with ADR1-L1-HA-TurboID resulted in only a small amount of biotinylated EDS1-ZZ-TEV-FLAG and PAD4-ZZ-TEV-FLAG (Figure 1, C). It is possible that the interactions between ADR1s and EDS1 or PAD4 require signals from upstream TIR/TNLs and the low levels of biotinylation of EDS1 and PAD4 by ADR1-L1-HA-TurboID are due to basal TNL activities in *N. benthamiana*. We therefore tested whether activation of TIR signaling can stimulate these interactions using the Arabidopsis TIR-only protein RBA1 (Response to HopBA1), which triggers EDS1/PAD4-dependent immune responses (Nishimura et al., 2017). Interestingly, RBA1 treatment greatly increased the biotinylation of EDS1-ZZ-TEV-FLAG and PAD4-ZZ-TEV-FLAG by ADR1-L1-HA-TurboID (Figure 1, C and D). However, the NADase-dead RBA1_E86A_ failed to induce a similarly enhanced biotinylation as WT RBA1 (Supplemental Figure S4), suggesting that the NADase activity from TIR signaling enhances the interactions between ADR1-L1 and the EDS1-PAD4 heterodimer. In contrast, when EDS1-ZZ-TEV-FLAG and SAG101-3FLAG were expressed with ADR1-L1-HA-TurboID, only EDS1-ZZ-TEV-FLAG, but not SAG101-3FLAG, was biotinylated by ADR1-L1-HA-TurboID (Supplemental Figure S5), supporting a specificity with EDS1-PAD4 dimer. Together, these data provide biochemical evidence to support the distinct EDS1/PAD4/ADR1s and EDS1/SAG101/NRG1s modules.

**Figure 1:**
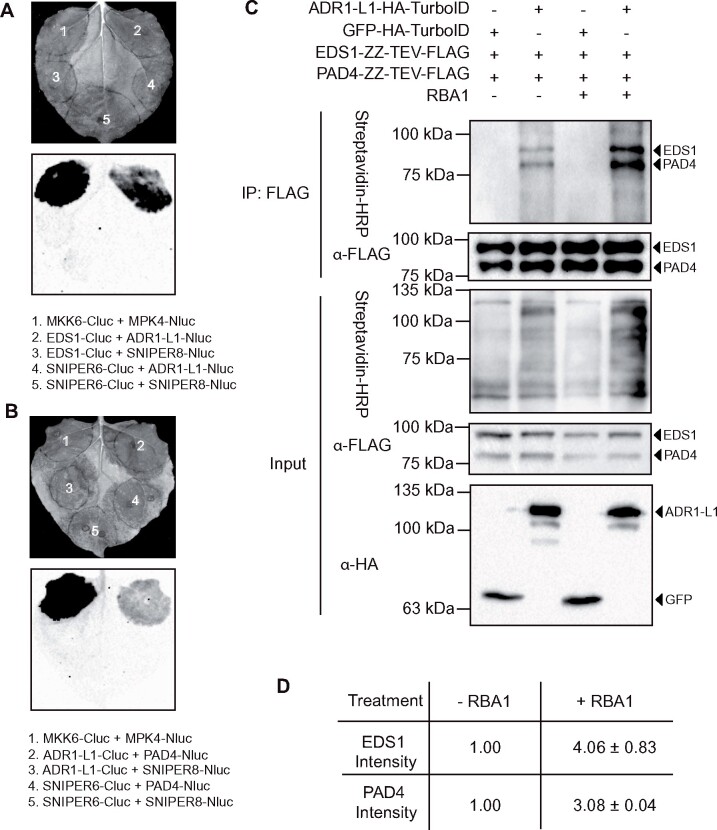
Activation of TIR signaling promotes the interaction between ADR1-L1 and the EDS1-PAD4 dimer. A and B, Interaction of ADR1-L1 with EDS1 (A) or PAD4 (B) as tested by SLC assay in *N. benthamiana*. The experiment was repeated three times with similar results. MPK4-Nluc and MKK6-Cluc were used as positive controls. The unpublished SNIPER6 and SNIPER8 are two immune-regulating E3 ligases isolated from *snc1*-influencing plant E3 ligase reverse (SNIPER) genetic screen, which were used as negative controls. C, IP and biotinylation of EDS1-ZZ-TEV-FLAG and PAD4-ZZ-TEV-FLAG by ADR1-L1-HA-TurboID in *N. benthamiana* without or with RBA1 pre-treatment. IP was carried out with anti-FLAG beads. The ZZ-TEV-FLAG-tagged proteins were detected using an anti-FLAG antibody. The HA-TurboID-tagged proteins were detected using an anti-HA antibody. The biotinylated proteins were detected using Streptavidin-HRP. Molecular mass marker in kilodaltons is indicated on the left. The experiment was repeated three times with similar results. D, Quantification of EDS1-ZZ-TEV-FLAG and PAD4-ZZ-TEV-FLAG band intensity of (C) in Streptavidin-HRP blot. The numbers represent the normalized ratio between the intensity of the IP-enriched biotinylated protein band and the corresponding IP-enriched protein band in FLAG blot ± sd (*n* = 3). Band intensity without RBA1 treatment was set to 1.

As oligomerization of NLRs is required for defense activation in both animal and plant systems (Jones et al., 2016) and *N. benthamiana* NRG1 self-associates (Qi et al., 2018), we tested whether ADR1-L1 can interact with itself. *In planta* association among ADR1-L1 proteins was observed in SLC (Figure 2, A). Such interaction was further confirmed with a co-IP experiment (Figure 2, B), indicating that ADR1-L1 self-associates. Likewise, RBA1 induction considerably increased the amount of ADR1-L1-3HA being pulled down (Figure 2, B and C). In contrast, RBA1_E86A_ was not able to increase ADR1-L1 self-association (Supplemental Figure S6), suggesting that the upstream TIR signaling is responsible for enhancing ADR1-L1 self-association. As CNL ZAR1 (HOPZ-ACTIVATED RESISTANCE 1) assembles into a pentameric complex (Wang et al., 2019), it will be interesting to determine whether ADR1-L1 also assembles into a pentamer upon activation.

**Figure 2:**
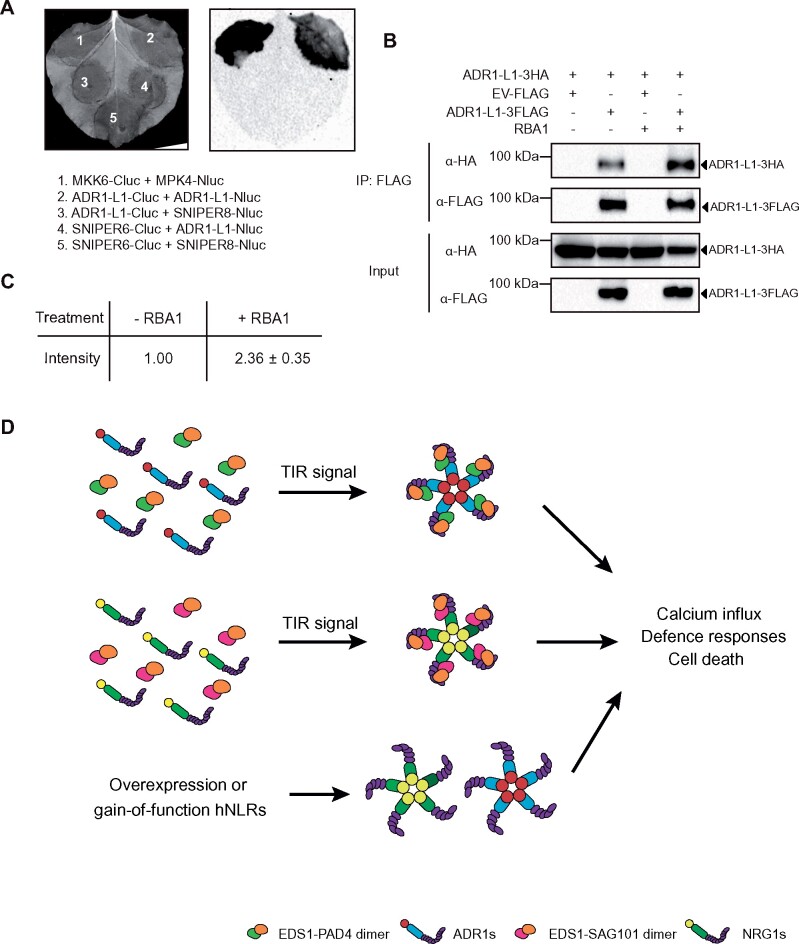
TIR signaling enhances the self-association of ADR1-L1. A, Self-association of ADR1-L1 as tested by SLC assay in *N. benthamiana*. The experiment was repeated three times with similar results. MPK4-Nluc and MKK6-Cluc were used as positive controls. SNIPER6 and SNIPER8 serve as negative controls. B, IP of ADR1-L1-3HA by ADR1-L1-3FLAG in *N. benthamiana* without or with RBA1 pre-treatment. IP was carried out with anti-FLAG beads. The 3FLAG-tagged proteins were detected using an anti-FLAG antibody. The HA-tagged proteins were detected using an anti-HA antibody. Molecular mass marker in kilodaltons is indicated on the left. The experiment was repeated three times with similar results. C, Quantification of ADR1-L1-3HA band intensity of (B) in the anti-HA blot. The numbers represent the normalized ratio between the intensity of the ADR1-L1-3HA protein band by FLAG pull-down and the IP-enriched ADR1-L1-3FLAG protein band in FLAG blot ± sd (*n* = 3). Band intensity without RBA1 treatment was set to 1. D, Working model of two defense modules upon activation of TIR signaling. TIR signaling leads to the generation of a product that can be perceived by either the EDS1-PAD4 or the EDS1-SAG101 dimers, which then triggers the assembly of EDS1-SAG101-NRG1 (Sun et al., 2021) and EDS1-PAD4-ADR1 complexes (current study), respectively, to activate defense responses. The formation of the ADR1 or NRG1 pentameric resistosome complexes may serve as Ca^2+^ influx channels, resulting in downstream immune activation and cell death (Jacob et al., 2021). Overexpression or auto-active gain-of-function versions of the hNLRs can trigger self-oligomerizations and Ca^2+^ channel formation without the requirement of the lipase-like proteins.

The N-terminally truncated NRG1C can associate with the EDS1-SAG101 dimer and interfere with the EDS1-SAG101-NRG1 module (Wu et al., 2021), implying that the part of NRG1 interacting with EDS1-SAG101 is likely through the C-terminal LRR and part of the NB domain. We therefore examined the EDS1-PAD4-ADR1 interactions with an N-terminally truncated ADR1. A construct overexpressing the truncated ADR1 similar to that of NRG1C (hereafter named as ADR1(1C)) was generated based on the protein sequence alignment (Supplemental Figures S7, S8, A) and was introduced into the *snc1* background, which contains a gain-of-function mutation in the *TNL SNC1* that results in ADR1s-dependent autoimmunity (Dong et al., 2016). Overexpression of *ADR1(1C)* partially suppressed *snc1*-mediated dwarfism and resistance to the oomycete pathogen *Hyaloperonospora arabidopsidis* (*H.a.*) Noco2 (Supplemental Figure S8, B and C). However, the transcript levels of *ADR1-L1* and *ADR1-L2* (Supplemental Figure S8, D and E) were not affected by *ADR1(1C)* overexpression (Supplemental Figure S8, F), excluding the possibility of suppression through gene silencing. Furthermore, when the HA-TurboID-ADR1(1C) was co-expressed with EDS1-ZZ-TEV-FLAG and PAD4-3FLAG, biotinylated EDS1-ZZ-TEV-FLAG and PAD4-3FLAG were detected (Supplemental Figure S9, A), suggesting that ADR1(1C) is in close proximity with EDS1 and PAD4. ADR1(1C) likely acts as a dominant-negative form to interfere with the EDS1-PAD4-ADR1 module. In agreement, addition of HA-TurboID-ADR1(1C) greatly reduced the SLC signals observed when EDS1-Cluc and ADR1-L1-Nluc were co-expressed (Supplemental Figure S9, B and C). Together, these data support that ADR1s likely associate with the EDS1-PAD4 dimer through their C-terminal NB-LRR region, although we cannot exclude the alternative explanation that the truncated ADR1 is in close proximity with EDS1/PAD4 via an interaction with endogenous full-length ADR1, rather than directly. Future structural and functional analyses are needed to resolve the detailed protein-protein interaction interfaces in the EDS1/PAD4/ADR1s module.

In summary, our study showed that activation of TIR signaling stimulates the interactions between ADR1-L1 and EDS1/PAD4 and self-association of ADR1-L1. Combining with findings from two other studies showing that TIR signaling promotes the interaction between NRG1s and EDS1/SAG101 (Sun et al., 2021; Qi et al., 2018), a conceptual model for the roles of EDS1/PAD4/SAG101 and the hNLRs is proposed (Figure 2, D). Upon recognition of pathogen effectors, TIR/TNL receptors are activated, leading to the generation of an NADase product(s), which is subsequently recognized by EDS1-PAD4 and EDS1-SAG101. Recognition of this signal molecule(s) is proposed to stimulate the interactions of EDS1-PAD4 and EDS1-SAG101 with the hNLRs, leading to self-association of hNLRs and formation of hNLR oligomeric complexes. In contrast, overexpression or auto-active versions of hNLRs are capable of self-association, leading to EDS1-independent defense activation (Qi et al., 2018; Wu et al., 2019; Supplemental Figure S10 and Figure 2, D). According to two recent reports (Bi et al., 2021; Jacob et al., 2021), similar to ZAR1, the hNLR oligomers can also form Ca^2+^ channels on the plasma membrane to activate downstream immune responses and cell death. Therefore, the hNLRs serve as receptors for the ligand-bound EDS1-PAD4 and EDS1-SAG101 heterodimers for TIR signaling. Upon activation, they may form ZAR1 resistosome-like pentameric rings that can serve as Ca^2+^ influx channels to turn on immune responses and cell death. Future investigations on the nature of the signaling molecule produced by the TIR NADase activity and how it interacts with the lipase-like proteins are needed for full understanding of the TIR signaling pathway in plants.

## Supplemental data

**Supplemental Figure S1.** The EDS1-PAD4-ADR1 module functions in parallel with the EDS1-SAG101-NRG1 module.

**Supplemental Figure S2.** ADR1-HA-TurboID, not ADR1-L1-HA-TurboID, causes cell death in *N. benthamiana*.

**Supplemental Figure S3.** The protein expressions in SLC assays in *N. benthamiana*.

**Supplemental Figure S4.** The NADase dead RBA1_E86A_ fails to enhance the association between ADR1-L1 with EDS1/PAD4.

**Supplemental Figure S5.** EDS1-ZZ-TEV-FLAG, but not SAG101-3FLAG, is biotinylated by ADR1-L1-HA-TurboID.

**Supplemental Figure S6.** RBA1_E86A_ cannot enhance ADR1-L1 self-association as with WT RBA1.

**Supplemental Figure S7.** Protein alignment of Arabidopsis ADR1-L1, ADR1-L2, ADR1, ADR1-L3, NRG1C, and NRG1C-type truncated ADR1 (ADR1(1C)).

**Supplemental Figure S8.** Overexpression of *ADR1*(*1C*) partially suppresses *snc1*-mediated dwarfism/autoimmunity.

**Supplemental Figure S9.** ADR1(1C) interacts with EDS1-PAD4 dimer.

**Supplemental Figure S10.** ADR1-3FLAG causes cell death in WT and *eds1 N. benthamiana*.

**Supplemental Table S1.** The list of primers used in this study

## Supplementary Material

kiab305_Supplementary_Data

## Notes

Z.W. and L.T.: data curation, validation, investigation, methodology, writing—original draft, and project administration. X.Liu: validation and methodology. Y.Z. and X.Li: conceptualization, data curation, formal analysis, supervision, funding acquisition, writing—original draft and revisions, and project administration. All authors reviewed the manuscript.

The author responsible for distribution of materials integral to the findings presented in this article in accordance with the policy described in the Instructions for Authors (https://academic.oup.com/plphys/pages/general-instructions) is: Xin Li (xinli@msl.ubc.ca).
