# Supplementary material for: TIR signal promotes interactions between lipase-like proteins and ADR1-L1 receptor and ADR1-L1 oligomerization
Source: Plant Physiol. 2021 Jul 2;187(2):681–6. doi: 10.1093/plphys/kiab305 (PMC8491023; doi:10.1093/plphys/kiab305)
Supplement: kiab305_Supplementary_Data [file kiab305_supplementary_data.pdf]

TIR signal promotes interactions between lipase-like proteins and ADR1-L1 receptor and ADR1-L1 oligomerization

Supplemental Figure S1. The EDS1-PAD4-ADR1 module functions in parallel with the EDS1-SAG101-NRG1 module.

Supplemental Figure S2. ADR1-HA-TurboID, not ADR1-L1-HA-TurboID, causes cell death in *N. benthamiana*.

Supplemental Figure S3. The protein expressions in SLC assays in *N. benthamiana*.

Supplemental Figure S4. The NADase dead $RBA1_{E86A}$ fails to enhance the association between ADR1-L1 with EDS1/PAD4.

Supplemental Figure S5. EDS1-ZZ-TEV-FLAG, but not SAG101-3FLAG, is biotinylated by ADR1-L1-HA-TurboID.

Supplemental Figure S6. $RBA1_{E86A}$ did not enhance ADR1-L1 self-association as with WT RBA1.

Supplemental Figure S7. Protein alignment of Arabidopsis ADR1-L1, ADR1-L2, ADR1, ADR1-L3, NRG1C and NRG1C-type truncated ADR1 (ADR1(1C)).

Supplemental Figure S8. Overexpression of *ADR1*(*1C*) partially suppresses *snc1*-mediated dwarfism/autoimmunity.

Supplemental Figure S9. ADR1(1C) interacts with EDS1-PAD4 dimer.

Supplemental Figure S10. ADR1-3FLAG causes cell death in WT and *eds1 N. benthamiana*.

Supplemental Table S1. The list of primers used in this study.

Fig. S1

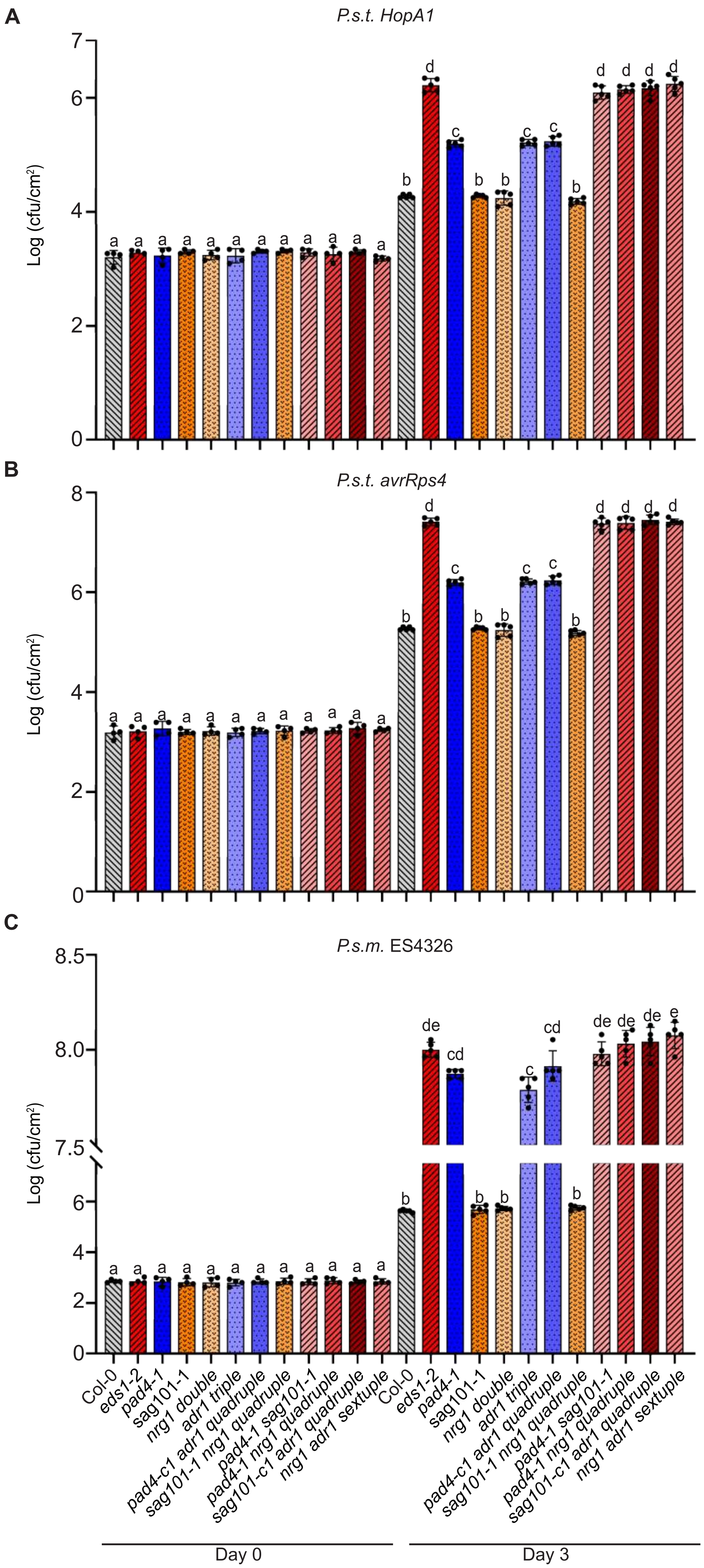

Supplemental Figure S1. The EDS1-PAD4-ADR1 module functions in parallel with the EDS1-SAG101-NRG1 module.

A-C. Growth of *P.s.t. HopA1* (A), *avrRps4* (B) and *P.s.m.* ES4326 (C) in four-week-old leaves of the indicated genotypes at 0 dpi (day post inoculation) and 3 dpi, with bacterial inoculum of OD_{600} = 0.0001. cfu, colony-forming unit. Statistical significance is indicated by different letters ($p < 0.01$, one-way ANOVA followed by Tukey's multiple comparisons test). Error bars represent means ± SD (n=4 for day 0, n=5 for day 3). Three independent experiments were carried out with similar results.

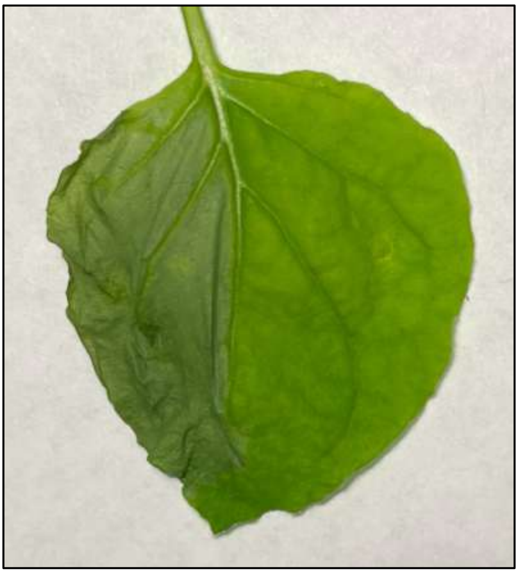

ADR1-HA-TurboID ADR1-L1-HA-TurboID

Supplemental Figure S2. ADR1-HA-TurboID, not ADR1-L1-HA-TurboID, causes cell death in *N. benthamiana*.

HR in the *N. benthamiana* leaves expressing ADR1-HA-TurboID (left) or ADR1-L1-HA-TurboID (right). Photos were taken at 36 hpi. Three independent experiments were carried out with similar results.

Fig. S3

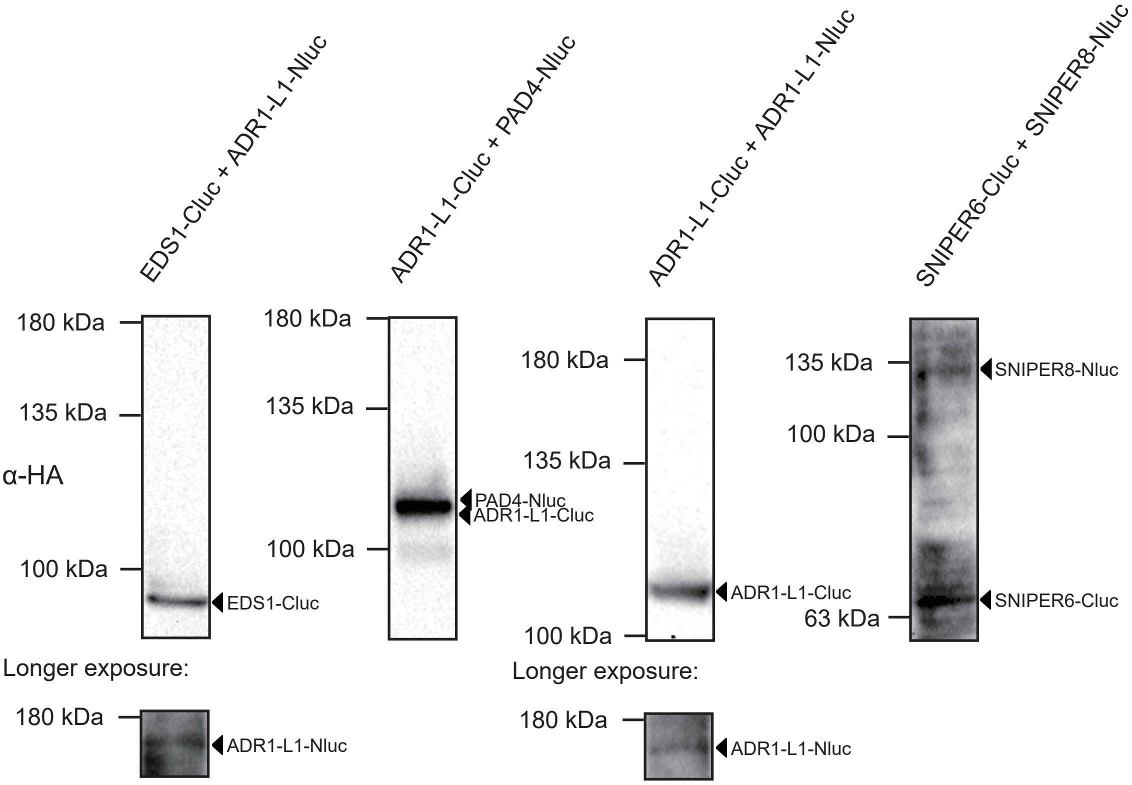

Supplemental Figure S3. The protein expressions in SLC assays in *N. benthamiana*.

Western blots showing the expression of EDS1-Cluc, ADR1-L1-Nluc, PAD4-Nluc, ADR1-L1-Cluc, SNIPER6-Cluc and SNIPER8-Nluc. Molecular mass marker in kiloDaltons is indicated on the left. The molecular weights of PAD4-Nluc (111.7 kDa) and ADR1-L1-Cluc (109 kDa) are too close to separate.

A

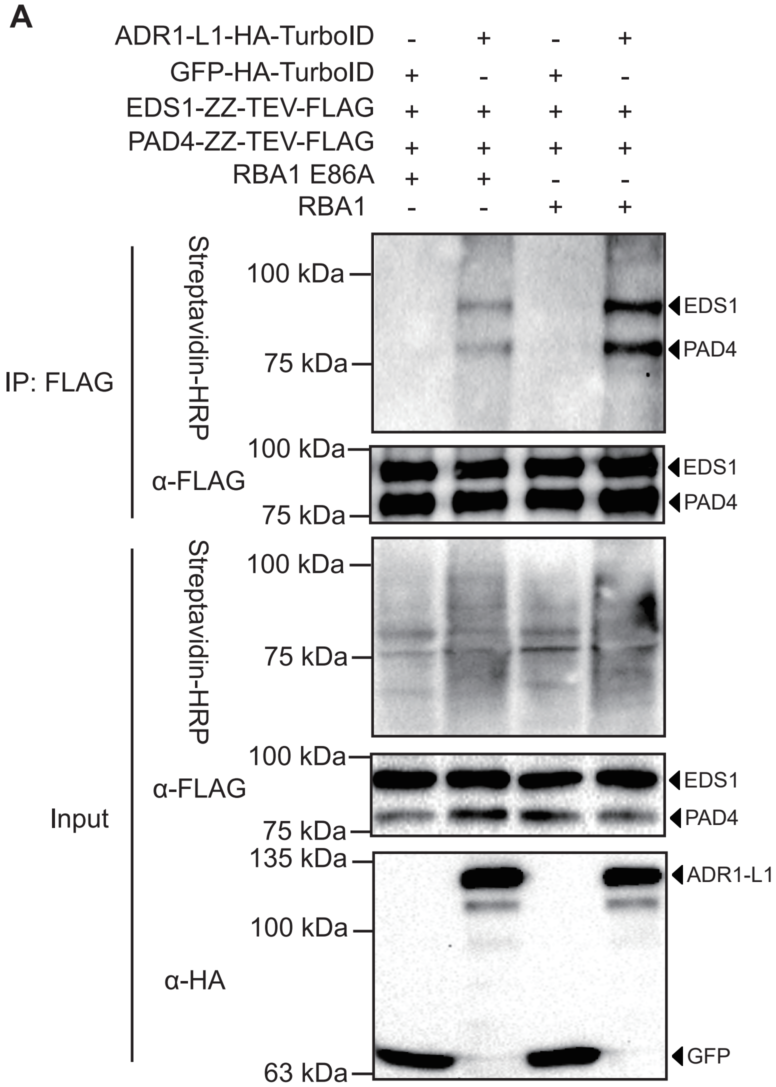

B

Treatment	+ RBA1 E86A	+ RBA1
EDS1 Intensity	1.00	3.65 ± 0.09
PAD4 Intensity	1.00	3.21 ± 0.21

Supplemental Figure S4. The NADase dead RBA1$_{E86A}$ fails to enhance the association between ADR1-L1 with EDS1/PAD4.

A. Immunoprecipitation and biotinylation of EDS1-ZZ-TEV-FLAG and PAD4-ZZ-TEV-FLAG by ADR1-L1-HA-TurboID in *N. benthamiana* with RBA1 or RBA1$_{E86A}$ pre-treatment. Immunoprecipitation was carried out with anti-FLAG beads. The ZZ-TEV-FLAG-tagged proteins were detected using an anti-FLAG antibody. The HA-TurboID-tagged proteins were detected using an anti-HA antibody. The biotinylated proteins were detected using Streptavidin-HRP. Molecular mass marker in kiloDaltons is indicated on the left. The experiment was repeated twice with similar results.

B. Quantification of EDS1-ZZ-TEV-FLAG and PAD4-ZZ-TEV-FLAG band intensity of (A) in Streptavidin-HRP blot. The numbers represent the normalized ratio between the intensity of the IP-enriched biotinylated protein band and the corresponding IP-enriched protein band in FLAG blot ± SD (n=2). Band intensity with RBA1$_{E86A}$ treatment was set to 1.

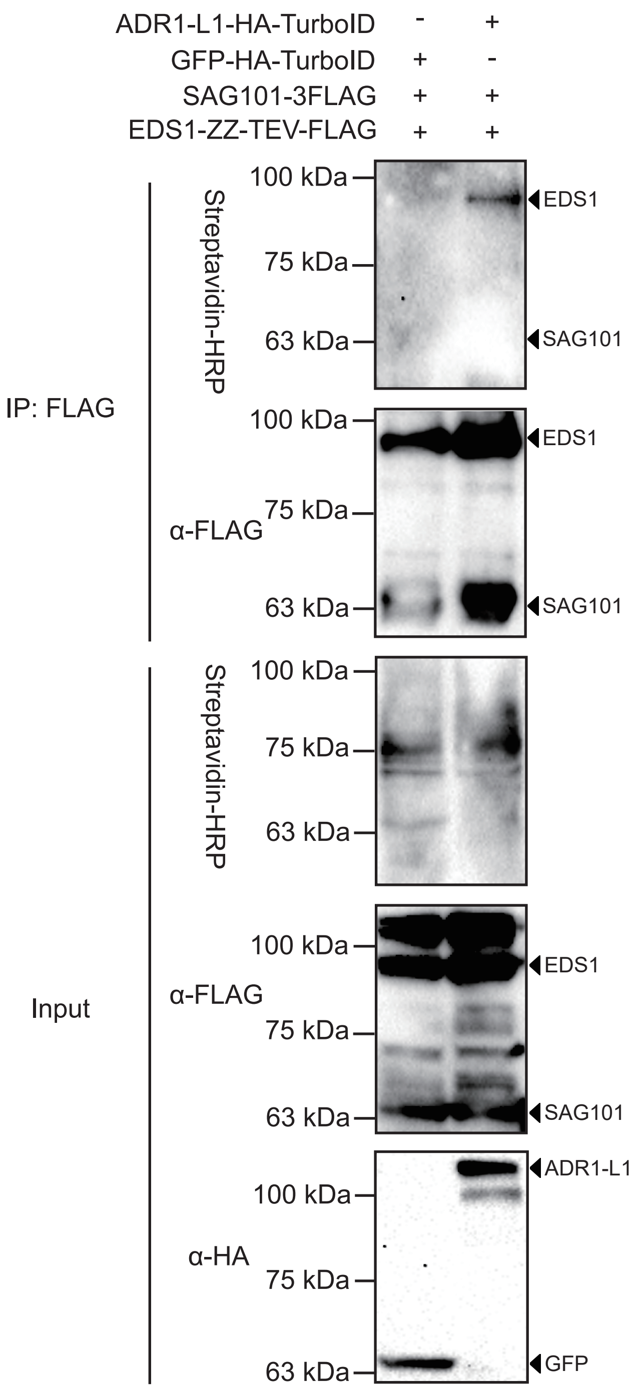

Supplemental Figure S5. EDS1-ZZ-TEV-FLAG, but not SAG101-3FLAG, is biotinylated by ADR1-L1-HA-TurboID.

A. Immunoprecipitation and biotinylation of EDS1-ZZ-TEV-FLAG and SAG101-3FLAG by ADR1-L1-HA-TurboID in *N. benthamiana*. Immunoprecipitation was carried out with anti-FLAG beads. The ZZ-TEV-FLAG and 3FLAG-tagged proteins were detected using an anti-FLAG antibody. The HA-TurboID-tagged proteins were detected using an anti-HA antibody. The biotinylated proteins were detected using Streptavidin-HRP. Molecular mass marker in kiloDaltons is indicated on the left. The experiment was repeated twice with similar results.

A

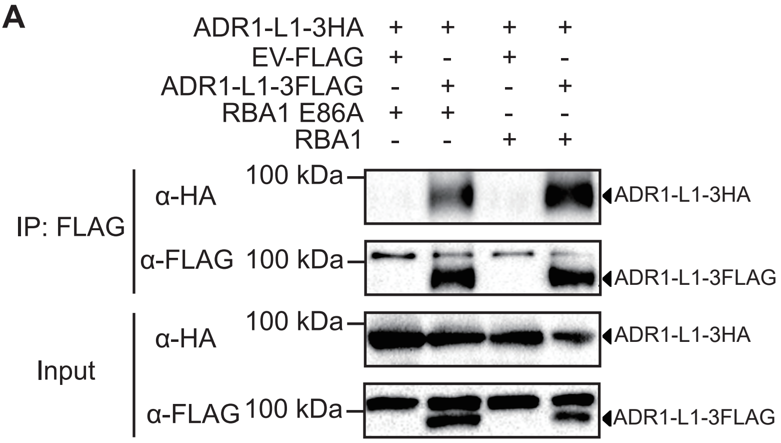

B

Treatment	+ RBA1 E86A	+ RBA1
Intensity	1.00	2.05 ± 0.45

Supplemental Figure S6. RBA1$_{E86A}$ did not enhance ADR1-L1 self-association as with WT RBA1.

A. Immunoprecipitation of ADR1-L1-3HA by ADR1-L1-3FLAG in *N. benthamiana* with RBA1 or RBA1$_{E86A}$ pre-treatment. Immunoprecipitation was carried out with anti-FLAG beads. The 3FLAG-tagged proteins were detected using an anti-FLAG antibody. The HA-tagged proteins were detected using an anti-HA antibody. Molecular mass marker in kiloDaltons is indicated on the left. The experiment was repeated twice with similar results.
B. Quantification of ADR1-L1-3HA band intensity of (A) in the anti-HA blot. The numbers represent the normalized ratio between the intensity of the ADR1-L1-3HA protein band by FLAG pull-down and the IP-enriched ADR1-L1-3FLAG protein band in FLAG blot ± SD (n=2). Band intensity without RBA1$_{E86A}$ treatment was set to 1.

Fig. S7

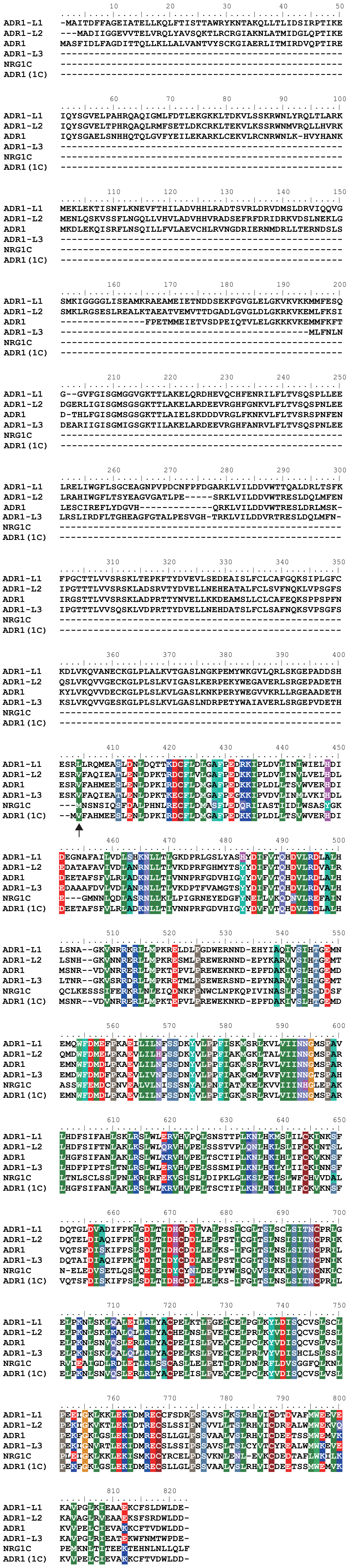

Supplemental Figure S7. Protein alignment of Arabidopsis ADR1-L1, ADR1-L2, ADR1, ADR1-L3, NRG1C and NRG1C-type truncated ADR1 (ADR1(1C)).

Protein sequences were obtained from TAIR. Arrow indicates the truncation location on ADR1

Fig. S8

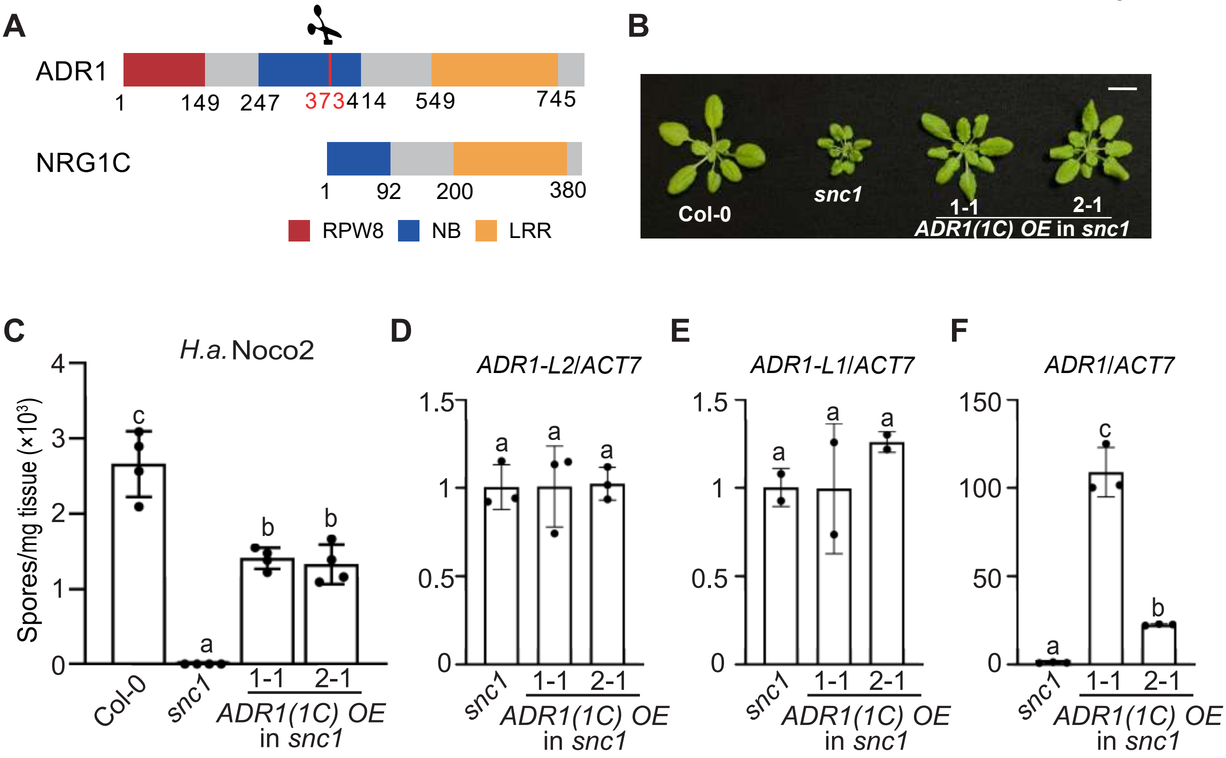

Supplemental Figure S8. Overexpression of *ADR1*(*1C*) partially suppresses *snc1*-mediated dwarfism/autoimmunity.

A. Protein domain diagram of NRG1C and ADR1. Numbers represent amino acid positions relative to the translation start sites. Scissor and red line indicate the truncation location on ADR1 for generating ADR1(1C).
B. Morphology of four-week-old soil-grown plants of Col-0, *snc1* and two independent transgenic lines of *ADR1(1C) OE* into *snc1* background.
C. Quantification of *H.a.* Noco2 sporulation in the indicated genotypes at 7 days post inoculation (dpi) with 10^5 spores per ml water. Statistical significance is indicated by different letters ($p <$ 0.01, one-way ANOVA followed by Tukey's multiple comparisons test). Bars represent means ± SE (n=4). Three independent experiments were carried out with similar results.
D-F. Expression levels of *ADR1-L2* (D), *ADR1-L1* (E) or *ADR1* (F) in the indicated genotypes as determined by RT-qPCR (reverse transcription quantitative PCR) (*snc1* serves as control whose transcript level was set at 1.0). The *ADR1* transcripts measured include the ones for both the native full-length *ADR1* and the truncated *ADR1(1C)* as the primers used for RT-qPCR can amplify both. Statistical significance is indicated by different letters ($p < 0.01$, one-way ANOVA followed by Tukey's multiple comparisons test). Bars represent means ± SE (n = 3 for D and F; n=2 for E). Two independent experiments were carried out with similar results.

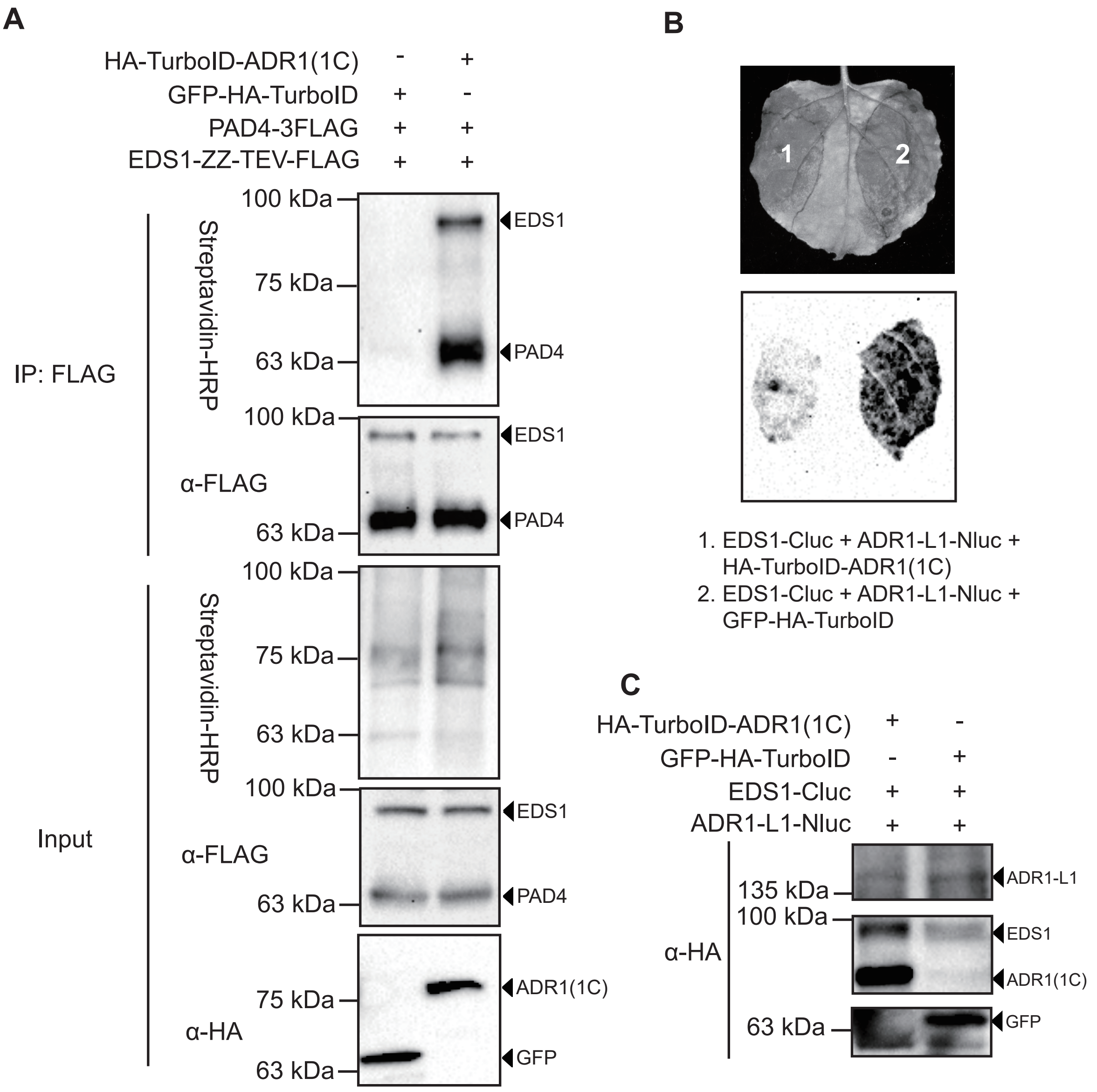

Supplemental Figure S9. ADR1(1C) interacts with EDS1-PAD4 dimer.

A. Immunoprecipitation and biotinylation of EDS1-ZZ-TEV-FLAG and PAD4-3FLAG by HA-TurboID-ADR1(1C) in *N. benthamiana*. Immunoprecipitation was carried out with anti-FLAG beads. The ZZ-TEV-FLAG and 3FLAG-tagged proteins were detected using an anti-FLAG antibody. The HA-TurboID-tagged proteins were detected using an anti-HA antibody. The biotinylated proteins were detected using Streptavidin-HRP. Molecular mass marker in kiloDaltons is indicated on the left. The experiment was repeated three times with similar results.
B. Interaction of ADR1-L1 with EDS1 as tested by split-luciferase complementation assay in *N. benthamiana* in the presence of HA-TurboID-ADR1(1C) (Left) or GFP-HA-TurboID (Right). The experiment was repeated three times with similar results.
C. Western blot showing the protein expression in (B). Molecular mass marker in kiloDaltons is indicated on the left.

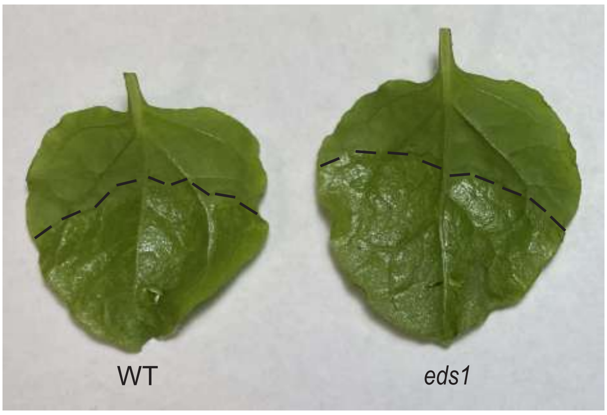

Supplemental Figure S10. ADR1-3FLAG causes cell death in WT and *eds1 N. benthamiana*.

HR in the *N. benthamiana* leaves expressing ADR1-3FLAG in WT (Left) or *eds1* (Right) *N. benthamiana*. Photos were taken at 36 hpi. Three independent experiments were carried out with similar results.

Table S1. The list of primers used in this study

Name	Sequence	Purpose
ADR1(1c)-KpnI-F	gcgcggtaccATGGTG TTTGCTCATATGGAAG	To generate pBASTA HATurboID ADR1(1C) and pBASTA ADR1(1C)
ADR1-SpeI(1C)-R	GGACTAGTTAATCGTCAAGCCAATCC	
EDS1-DraIII-F	cggcggCACTCAGTGATGGCGTTTGAAGCTC	To generate pCambia 1300 Cluc-EDS1
EDS1-DraIII-R	cggcggCACATGGTGTCAGGTATCTGTTATTTCATCC	
PAD4-SfiI-F	CCCGGCCGTCAAGGCCaATGGACGATTGTCGATT	To generate pCambia 1300 PAD4-Nluc
PAD4-SfiI(ns)-R	GCCGGCCCATGAGGCCAGTCTCCATTGCGTCAC	
PAD4-KpnI-F	gcgcggtaccATGGACGATTGTCGATT	To generate pBASTA PAD4-3FLAG
PAD4-SpeI(ns)-R	GGACTAGTAGTCTCCATTGCGTCAC	
ADR1-L1-KpnI-NF	gcgcggtaccATGGCCATCACCGATTT	To generate pBASTA ADR1-L1-HATurboID
ADR1-l1-SpeI(ns)-NR	gactagtTTCGTCAAGCCAGTCTAG	
ADR1-L1-KpnI-NF	gcgcggtaccATGGCCATCACCGATTT	To generate pCambia 1300 ADR1-L1-FLAG
ADR1-l1-SmaI(ns)-R	tcccccgggTTCGTCAAGCCAGTCTAGG	
ADR1-l1-KpnI-F	gcgcggtaccCATGGCCATCACCGATTT	To generate pCambia 1300 ADR1-L1-Nluc and pC1300 ADR1-L1-Cluc
ADR1-l1- Sal(ns)-R	CGCGTCGACTTCGTCAAGCCAGTCTAGG	
ADR1-L2 RT-F new	CTTGTGAAAGATCCAAGGTT	L2 RT
ADR1-L2 RT-R new	TGAGTCATTTCTCCTGTGT	
ADR1-L1 RT-F	GTGTGACAAATCAACAAGG	L1 RT
ADR1-L1 RT-R	GATCTGGTTAAGCAGGTT	
ADR1 RT-F new	ATAGTGAACAATCCGAGGTT	ADR1 RT
ADR1 RT-R new	TTTCATCCATTTCCCCTGT	

Supplemental Methods

Plant growth conditions

A. thaliana and *N. benthamiana* plants were grown at 22 ºC under a long day condition of 16 hr light/8 hr dark regime.

Construction of plasmids

Overexpression and HA-TurboID tagged *ADR1*(*1C*) constructs were made in *pBASTA* and *pBASTA-N-HA-TurboID* vectors, respectively. *EDS1*, *PAD4* and *ADR1-L1* were cloned into *pCambia 1300-Cluc* or *-Nluc*. *PAD4* was cloned into *pBASTA-3FLAG*. *ADR1-L1* was cloned into *pBASTA-HA-TurboID*, *pCambia1300-3FLAG* and *pCambia1300-3HA*. All primers used are listed in Supplemental Table 1. *pCambia1305 EDS1-ZZ-TEV-FLAG*, *pCambia1305 PAD4-ZZ-TEV-FLAG*, *pBASTA GFP-HA-TurboID* were described in previous studies (Wu et al., 2021). The CRISPR/Cas9 constructs for knocking out *PAD4* and *SAG101* were described in previous studies (Wu et al., 2021).

A. thaliana transformation

The binary constructs were introduced into *Agrobacterium tumefaciens* GV3101 by electroporation and subsequently transformed into *A. thaliana snc1* background by the floral-dipping method (Clough & Bent, 1998). Transformants were selected on soil by spraying BASTA (Glufosinate ammonium). The co-segregation analysis was then performed with the transformants in T_2 generations to make sure the suppression phenotypes were due to the transgene overexpression.

Pathogen infection assay

Pathogen infection assays were carried out as described previously (Li et al., 2001). In brief, two-week-old soil-grown seedlings were sprayed with 10^5 *H.a.* Noco2 conidia spores per ml water. The plants were transferred to a humid chamber at 18 °C of 12 hr light/12 hr dark. After 7 days, sporulation was quantified using a hemocytometer. For bacterial infections, four-week-old plants were infiltrated with bacterial solution at designated concentrations using a blunt-end syringe. Leaf discs were collected and ground at 0- and 3-day post infiltration. Colony-forming units (CFU) were calculated after incubation on LB plates with appropriate antibiotic selection.

TurboID-based proximity labeling in *N. benthamiana*

TurboID-based proximity labeling assay was performed as described previously (Wu *et al.*, 2020; Zhang *et al.*, 2019). In brief, *N. benthamiana* leaves were infiltrated with agrobacteria containing *HA-TurboID*, *3FLAG* and *ZZ-TEV-FLAG* tagged constructs. At 24 hpi, agrobacteria expressing RBA1 constructs as well as the biotin were infiltrated. A similar amount of leaf samples was harvested at 36 dpi, followed by immunoprecipitation and western blot analysis.

Co-immunoprecipitation, and western blot analysis

Co-immunoprecipitation assay was performed as previously described (Wu et al., 2020). In brief, about 2.0 g *N. benthamiana* leaves expressing the indicated proteins were harvested at 36 hpi and ground into fine powder with liquid nitrogen. Extraction buffer contains 25 mM Tris-HCl pH 7.5, 300 mM NaCl, 5 mM $MgCl_2$, 0.15% Nonidet P-40, 10% Glycerol, 1 mM PMSF, 1× Protease Inhibitor Cocktail (Roche; Cat. #11873580001), and 10 mM DTT. For testing the interaction between ADR1-L1 with EDS1-PAD4 and the self-association of ADR1-L1, 20 µM dATP and 5 mM $CaCl_2$ were added to the extraction buffer to be able to consistently observe the interactions, as NLRs are ATPases and the maintenance of their oligomeric form requires the presence of ATP/dATP (Wang et al., 2019). The FLAG-tagged proteins were immunoprecipitated using 20 µl M2 beads (Sigma Cat. #A2220) and biotinylation was detected by Streptavidin-HRP (Abcam Cat. # ab7403). The anti-HA antibody was from Roche (Cat. #11867423001). The anti-FLAG antibody was from Sigma (Cat. #F1804). Protein abundance was quantified using ImageJ (https://imagej.nih.gov/ij/).

Split luciferase complementation assay

Agrobacteria expressing the luciferase C-terminus (C-luc) and N-terminus (N-luc)-fused proteins were co-infiltrated into four-week-old *N. benthamiana* leaves, and the infiltrated leaves were treated with 1 mM luciferin after 48 hpi. Each bacterial strain was diluted to a final concentration of OD_{600} = 0.2.

Expression analysis

About 0.05 g of four-week-old soil-grown plant tissue was collected, and RNA was extracted using an RNA isolation kit (Bio Basic; Cat#BS82314). ProtoScript II reverse transcriptase (NEB; Cat#B0368) was used to generate cDNA. Real-time PCR was performed using a SYBR premix kit (TaKaRa, Cat#RR82LR). The primers used are listed in Supplemental Table 1.

Statistical analysis

Statistical analysis was carried out with one-way ANOVA followed by Tukey's post hoc test. The Scheffé multiple comparison was applied for testing correction. Normality test for all data was done in SPSS. Statistical significance was indicated with different letters. *p* values and sample numbers (n) were claimed in figure legends.

References

Clough, S. J., & Bent, A. F. (1998). Floral dip: a simplified method for Agrobacterium-mediated transformation of *Arabidopsis thaliana*. *The plant journal*, *16*(6), 735-743.

Li, X., Clarke, J. D., Zhang, Y., & Dong, X. (2001). Activation of an EDS1-mediated R-gene pathway in the snc1 mutant leads to constitutive, NPR1-independent pathogen resistance. *Molecular plant-microbe interactions*, *14*(10), 1131-1139.

Wu, Z., Tong, M., Tian, L., Zhu, C., Liu, X., Zhang, Y., & Li, X. (2020). Plant E3 ligases SNIPER 1 and SNIPER 2 broadly regulate the homeostasis of sensor NLR immune receptors. *The EMBO journal*, *39*(15), e104915.

Wu, Z., Tian, L., & Li, X. (2021). N-terminally truncated helper NLR NRG1C antagonizes immunity mediated by its full-length neighbors NRG1A and NRG1B. *bioRxiv*.

Zhang, Y., Song, G., Lal, N. K., Nagalakshmi, U., Li, Y., Zheng, W., ... & Dinesh-Kumar, S. P. (2019). TurboID-based proximity labeling reveals that UBR7 is a regulator of N NLR immune receptor-mediated immunity. *Nature communications*, *10*(1), 1-17.
